# Regulatory Mechanism of Carbon Fractions and Microbial Metabolism on Organic Carbon Turnover in Saline–Alkali Soils

**DOI:** 10.3390/biology15161335

**Published:** 2026-08-07

**Authors:** Jinfeng Wang, Fang Gao, Ziyuan Du, Yinheng Fan, Yaling Zan, Jia Li

**Affiliations:** College of Life Sciences, Yuncheng University, Yuncheng 044000, China

**Keywords:** saline–alkali soil, SOC, organic carbon fraction, CUE, microbial community structure

## Abstract

Soil salinization is a serious threat to agricultural productivity and organic carbon cycling; however, the mechanisms by which carbon fractions and microbial metabolism regulate organic carbon sequestration remain unclear. This research investigated the effects of four sites (S1–S4) along a salinity gradient on microbial carbon metabolism, carbon fractions (particulate organic carbon, POC; mineral-associated organic carbon, MAOC), and bacterial and fungal communities in a typical saline–alkali soil. The results showed that, as the salt–alkali level increases, microbial growth, respiration, and carbon utilization efficiency (CUE) all exhibit a gradually decreasing trend, with CUE declining by 34.0% at S4 relative to S1. CUE was strongly negatively correlated with the soil pH, EC, and soluble salts, but showed a logarithmic rise with SOC, reaching a maximum of 0.47 near 8 g kg^−1^. Both POC and MAOC decreased alongside the salt–alkali level, the proportion of MAOC in SOC (52.04–55.02%) was significantly higher than that of POC (44.98–47.96%), and there was a significant positive linear correlation with SOC and CUE. Under high salinity, the 16S rRNA gene copy number decreased by up to 28.8%, while the ITS copy number remained stable. As the salt and alkali levels increased, bacterial *Proteobacteria* and *Actinobacteriota* showed an increasing trend, whereas fungal communities remained dominated by *Ascomycota*. Furthermore, correlation analyses revealed that bacterial *Acidobacteriota* and *Proteobacteria*, and fungal *Basidiomycota* and *Chytridiomycota* play crucial roles in carbon cycling. Random forest modeling (RFM) and partial least squares path modeling (PLS-PM) also revealed that saline–alkali soil properties strongly affect 16S rRNA gene abundance and microbial respiration; carbon fractions and CUE exerted a strong positive direct effect on SOC, whereas microbial respiration had a negative effect. These findings demonstrate that saline–alkali stress restricts SOC sequestration primarily by weakening microbial activity and community function, with CUE playing only an indirect role. In conclusion, enhancing the POC and MAOC pools and improving the microbial carbon turnover efficiency may be more effective strategies for mitigating carbon loss and sustaining SOC stocks in saline–alkali soils.

## Simple Summary

Soil salination seriously threatens sustainable agriculture and soil organic carbon storage. Therefore, identifying the key limiting mechanisms of soil organic carbon under high-salt and alkali conditions is crucial. Most scientists working in this field aim to determine how soil microbes and their metabolism affect organic carbon turnover and stabilization under salt–alkali stress. Our study showed that increased salinity and alkalinity inhibited microbial activity and reduced the carbon use efficiency, which led to a drop in particulate organic carbon and mineral-associated organic carbon, ultimately lowering the overall soil organic carbon. Bacteria and fungi play important roles in the carbon cycle, and their community composition changes with salinity levels, with bacteria being more sensitive to salt–alkali stress. Salt stress mainly reduces soil carbon by inhibiting microbial respiration and decreasing organic carbon fractions. Boosting the organic carbon content and enhancing the microbial carbon use efficiency are potential strategies to slow down carbon loss in salt-affected soils and maintain soil fertility. This study is valuable for sustainable development in agricultural areas threatened by salinization and provides scientific and reasonable recommendations for fertilizer management and microbial agent production based on the microbial turnover characteristics of salty–alkali soils.

## 1. Introduction

Global agriculture is currently facing a major challenge due to soil salinization. Data show that over 8 × 10^8^ ha of farmland are affected by saline–alkali soil (FAO 2020), which severely restricts agricultural production and sustainability. The high salinity and pH of saline–alkali soil lead to lower soil organic carbon (SOC) content, and SOC storage continues to decrease as global saline–alkali soil expands [1]. The lower SOC content in saline–alkali soils may be attributed to (1) limited plant productivity, resulting in a decrease in the source or input of organic carbon [2]; (2) high salt ions and pH, which can alter the physical and chemical environment of the soil, thereby disrupting the protective mechanism of SOC [3,4]; (3) limited microbial activity and the simplistic community structure lead to a higher rate of SOC mineralization compared to synthesis and accumulation [5]. These factors, or their synergistic effects, jointly contribute to the lower and continuously decreasing organic carbon content in saline–alkali soils. Previous studies primarily focus on the sources of SOC and how its stability in saline–alkali soils changes with external carbon inputs, ignoring the influence of microorganisms and their carbon metabolism on organic carbon sequestration under salt and alkali stress. Microbial metabolism is the key process determining how exogenous carbon inputs are ultimately transformed into the soil’s own organic carbon; high salinity and pH generally inhibit microbial metabolic activity, slowing soil carbon cycling in saline–alkali soil [6]. Therefore, elucidating the carbon sequestration mechanisms through microbial metabolism is crucial for the sustainable development of saline–alkali soils.

Soil microorganisms are the key regulators of organic carbon transformation and stabilization [7]. Soil salinity and alkalinity may adversely affect soil microorganisms. Salinization can exert detrimental effects on soil microorganisms directly by inducing elevated osmotic stress and specific ion toxicity [8], or it can indirectly regulate microbial activity by reducing SOC content and plant residue inputs [9]. Soil salinity significantly influences the composition of soil microbial communities [10]. Microbial diversity tends to decrease with increasing soil salinity, while community dissimilarity amplifies alongside greater differences in salinity levels [11]. The fungi-to-bacteria ratio generally declines as the soil salinity increases, indicating that fungi are more susceptible to salinity stress than bacteria [12,13]. However, recent studies have revealed that, although bacterial diversity declines sharply with increasing salinity, the fungal Shannon diversity tends to remain stable [14]. Meanwhile, salinity selectively enriches halotolerant taxa such as *Proteobacteria* and *Bacteroidota* [15]. Moreover, with increasing salinity, bacterial community assembly shifts from stochastic to deterministic processes, with homogeneous selection becoming dominant [16], while the contribution of microbial necromass carbon to SOC decreases substantially under salt stress [17]. Generally, the response of the microbial carbon fixation capacity to salinity depends on the environmental background, the source of the salinity, and the microbial tolerance level. Although there is already an understanding of the effects of salt–alkali stress on soil microbial communities and function [18], the variation in saline–alkali soils remains poorly understood for microbial communities with specific carbon fixation functions or those adapted to saline–alkali stress. Geographically, the formation of saline–alkali land is a continuous succession process influenced by multiple factors, and its distribution shows significant spatial heterogeneity. However, the impacts of different degrees of salinity and alkalinity on the biological fractions of farmland soil ecosystems that affect the SOC pool have not received sufficient attention. In contrast, studies on forests, grasslands, and other ecosystems have consistently shown that the soil environment and the formation of biological communities are closely linked at the spatial scale (such as altitude, climate zones, etc.) [19]. The unique characteristics of saline–alkali soils have led to adaptive changes in soil microbial communities at both the individual and population levels, resulting in a distinctive ecological structure [10,20]. Moreover, as salinization intensifies, local microorganisms face pressure from environmental and invasive competitors, and the establishment of new groups and the integration of functions are crucial for success [21]. However, there is currently a lack of research exploring the differences in microbial community structure and their carbon metabolic functional potential in saline–alkali soils.

Salinization can also alter SOC fractions such as particulate organic carbon (POC), mineral-associated organic carbon (MAOC), and microbial biomass carbon (MBC), thereby regulating the SOC storage. POC is primarily derived from the decomposition products of plant-derived polymers and is highly susceptible to anthropogenic disturbances [22,23]. In contrast, MAOC is mainly composed of microbial byproducts, owing to its association with soil minerals, and resists rapid decomposition, representing a relatively stable organic carbon fraction [24]. Compared to the total SOC, POC and MAOC serve as more sensitive and accurate indicators for assessing changes in the organic carbon pools of saline–alkali soils. Moreover, MBC refers to the carbon contained within the living microbial community that drives decomposition. In stressful environments such as saline–alkali soils, MBC levels directly reflect the survival status and activity of the microbial community. Therefore, changes in organic carbon fractions in saline–alkali soils are crucial for understanding the stability and turnover mechanisms of organic carbon in such soils.

Microbial carbon utilization efficiency (CUE) is an important indicator reflecting the changes in soil organic carbon balance driven by microorganisms [25]. It is defined as the ratio of the organic carbon required for microbial growth to the total carbon absorbed [26]. Higher microbial CUE not only increases soil carbon sequestration by allocating a large amount of carbon to microbial residues but also reduces the carbon transferred and used for microbial respiration [27]. Conversely, higher carbon emissions and lower carbon conversion to biomass lead to lower microbial CUE [28]. Microbial CUE may vary significantly due to environmental factors, such as soil properties, carbon stability (carbon fractions), and the microbial community composition [29,30]. Soil properties affect microbial CUE by altering the microbial community structure and substrate utilization. Moreover, soil organic carbon fractions are key factors affecting microbial CUE. A previous study demonstrated a negative link between persistent mineral-associated organic carbon (MAOC) and CUE, as high-quality litter, which stimulates microbial growth and the production of extracellular enzymes, can accelerate the decomposition of soil organic carbon [31]. Meanwhile, changes in POC and MAOC can regulate the substrate utilization efficiency and then affect the soil C cycle [32]. Our previous research also revealed that, as the nutrient content increased, CUE actually decreased in coal mine reclaimed soil, and this was regulated jointly by soil nutrients, organic carbon fractions, and the microbial community composition [33]. However, for saline–alkali soils, apart from the relatively low carbon input, the low carbon utilization rate might be a microscopic reason for the low SOC content. Therefore, clarifying the differences in microbial CUE and related carbon metabolic mechanisms in saline–alkali soils can provide theoretical support for strategies to increase organic carbon in such soils.

Therefore, this study focuses on the relatively low organic carbon content in saline–alkali soils and elucidates how microbial carbon metabolism regulates organic carbon across different salt–alkali levels. We selected the farmland around the salt lake in Yuncheng City, Shanxi Province, China as typical saline–alkali farmland in an arid agricultural area. The planting system in this area is a rotation of wheat and maize, with maize straw returned to the soil during the wheat season, and all above-ground crop residues were removed during the maize season. We hypothesize that an increase in salinity will inhibit the carbon utilization efficiency (CUE) of soil microorganisms and alter the community composition, thereby affecting the distribution of organic carbon between mineralization and stabilization; moreover, the relative importance of abiotic and biotic factors in influencing soil organic carbon will change with the variation in the stress gradient. To test these hypotheses, we systematically investigated the microbial mechanisms underlying the differences in SOC turnover under various salt–alkali levels. The main objectives are as follows: (i) to study the different characteristics of the organic carbon fractions, microbial growth, respiration, CUE, and community structure in soils with different salt–alkali levels; (ii) to clarify the interrelationship between microbial C turnover and these environmental factors; and (iii) to quantify the influences of soil nutrients, organic carbon fractions, the microbial community composition, and microbial carbon turnover on SOC in saline–alkali soils and thereby reveal the mechanism by which microbial metabolism regulates organic carbon sequestration.

## 2. Materials and Methods

### 2.1. Study Area and Soil Sampling

The research areas are located in Yuncheng City (110°12′~111°41′ N; 34°48′~35°30′ E), in the south of Shanxi Province, China. It has a warm temperate continental monsoon climate, with plenty of sunshine and a small temperature difference between day and night. The average annual temperature and precipitation are 9.5 °C and 532.8 mm, respectively; the rainfall is mainly concentrated from July to September; the average annual evaporation is about 1768 mm; and the frost-free period is about 169 days. The farmland follows the conventional cropping system of wheat and maize rotation in this area. The soil type is characterized by silty loam and belongs to calcareous cinnamon soil.

**Figure 1:**
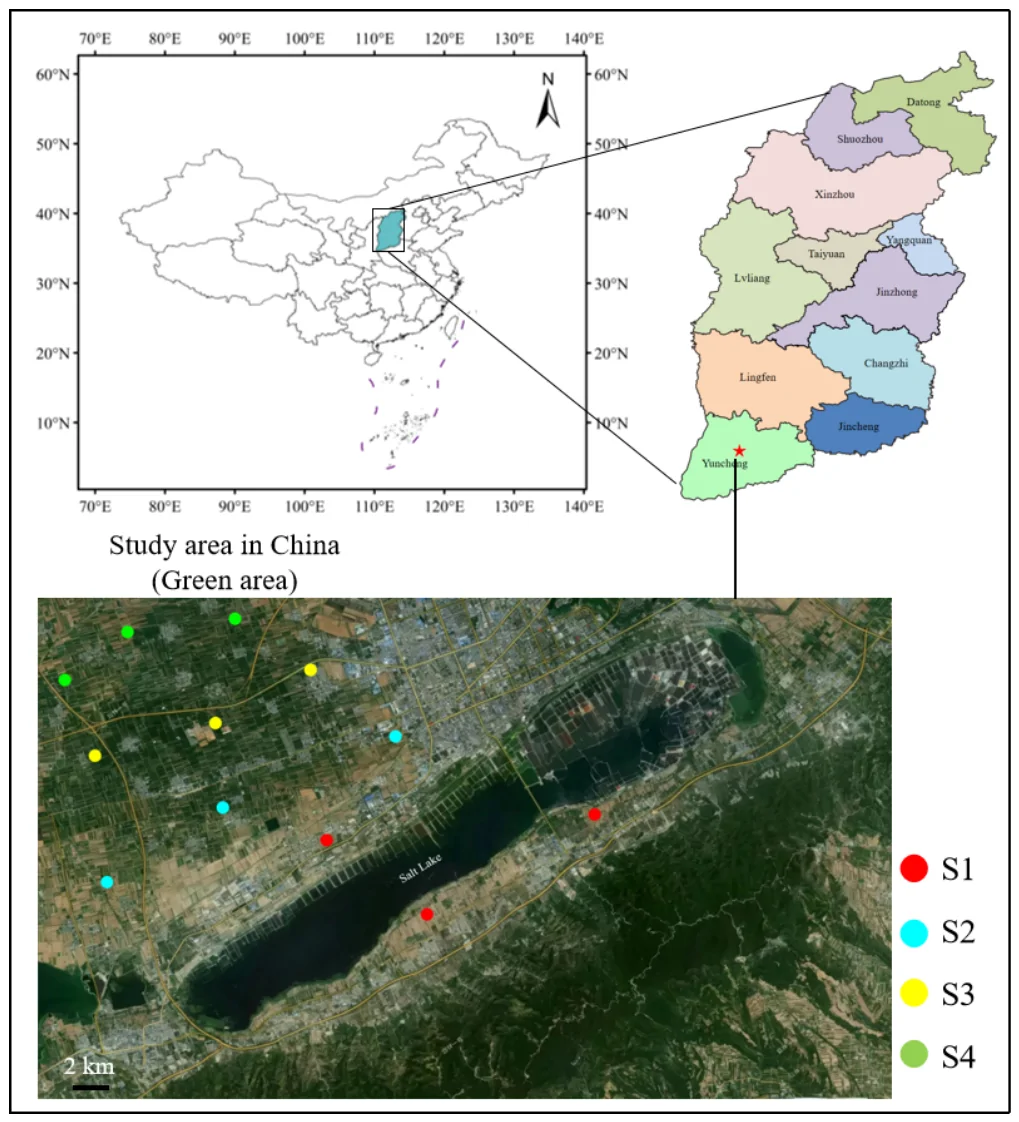
Location of study area and sample site distribution.

Soil samples were collected in October 2025 from the surrounding area of Yuncheng Salt Lake, known as the “China Dead Sea”—a typical inland sulfate salt-type lake. The agricultural land in this area is largely covered by saline–alkali soil. The location of the study area and sample site distribution are presented in Figure 1. The base maps of China and Shanxi Province used in this study were sourced from the National Geographical Information Public Service Platform of China (https://www.tianditu.gov.cn/; http://bzdt.ch.mnr.gov.cn/, accessed on 22 July 2026, map approval number: GS(2024)0650). The sampling area was located within a rectangular region extending approximately 20 km from east to west and 5 km from north to south, centered on the salt lake. Based on preliminary measurements of soil salinity, the sites were divided from the lakeside outward into four concentric zones (S4~S1) representing different salinization levels. Three representative farmland sites were selected within each salinization zone, and 12 typical farmland sites were established. At each sampling site, five subsamples were randomly collected from a depth of 0–20 cm within seven days after maize harvest and then homogenized to form one composite soil sample; a total of 12 soil samples were collected. All soil samples were sieved through a 2 mm mesh and stored at 4 °C until further analysis.

### 2.2. Soil Physicochemical Properties

The basic soil properties were determined using conventional methods [34]. The soil bulk density (BD) was measured using the ring knife method. The soil pH, Ec, and Eh (soil/water ratio = 1:2.5) were measured, respectively, using a pH meter, conductivity meter, and intelligent portable redox potentiometer. The soluble SO_4_^2−^, Ca^2+^, and Mg^2+^ in the soil were determined by the indirect complexometric titration method using EDTA; Na^+^ was determined by flame photometry; and Cl^−^ was determined by the silver nitrate (AgNO_3_) titration method. The total soluble salt ion content was calculated based on the sum of the above ions. Soil organic carbon (SOC) was determined by the potassium dichromate volumetric method, and total nitrogen (TN) was determined by the semimicro-Kjeldahl method. The basic physical and chemical properties of the soil at the four sampling sites are shown in Table 1. Overall, as the salinity levels increased, the soil BD, pH, Ec, Eh, and total and individual salt ion content all showed a gradual increase, while the soil nutrients (SOC, TN, and SOC/TN) showed a gradually decreasing trend from S1 to S4.

### 2.3. Soil Organic Carbon Fractions

Soil particulate and mineral-associated organic carbon (POC and MAOC) were determined using the sodium hexametaphosphate extraction method [35]. Briefly, 6.0 g of soil was placed in a 50 mL plastic bottle and mixed with 30 mL of 5 g L^−1^ sodium hexametaphosphate solution. The suspension was shaken for 18 h to achieve complete dispersion. The dispersed sample was then rinsed through 250 μm and 53 μm sieves with deionized water until the runoff was clear. The material retained on the 53 μm sieve (53–250 μm fraction) and that passing through (<53 μm) were collected separately, oven-dried at 60 °C, and weighed. The organic carbon content of each fraction was determined, and the POC and MAOC concentrations were calculated by multiplying the respective carbon concentrations by the proportion of each fraction in the bulk soil.

### 2.4. Microbial Growth, Respiration, and CUE

The ^18^O-labeled H_2_O method was used to determine the microbial carbon use efficiency (CUE), microbial growth, and respiration [28,30]. The detailed culture and determination procedures were as follows. Prior to formal incubation, in order to simulate field conditions and activate the microbial communities, fresh soil samples were pre-incubated at 25 °C in darkness for 7 days. After pre-incubation, the soil water content was determined; then, MBC was measured with 36 g soil, and duplicates of each soil sample (0.5 g) were placed in a 2 mL spiral sample bottle. Half of the soil duplicates were injected with ^18^O-H_2_O (98 atoms% ^18^O), and the final water atom% ^18^O was adjusted to 20. The other half were injected with ^16^O-H_2_O in the same volume as the ^18^O-H_2_O added during the treatments. Subsequently, the spiral bottle was exchanged for a 50 mL headspace bottle, which was capped, rinsed with CO_2_-free air for 5 min, and incubated at 25 °C for 24 h. After 24 h, CO_2_ production in the 50 mL headspace bottle was measured using a gas chromatograph (Agilent 7890, Agilent Technologies Inc., Santa Clara, CA, USA). In addition, soil microbial DNA was extracted using the FastDNA Spin Kit for Soil (MP Biomedicals, Irvine, CA, USA), and the Qubit dsDNA BR Assay Kit (Invitrogen, Carlsbad, CA, USA) was used to determine the DNA concentration. The remaining DNA extract was dried in silver capsules at 60 °C for 8 h to remove moisture. Finally, the O content (μg) and ^18^O abundance (atom%) were determined using a stable isotope ratio mass spectrometer (Thermo Delta V plus from Thermo Fisher, Bremen, Germany; EA2000, Thermo Fisher, Dreieich, Germany).

During the incubation period, the total DNA produced (DNA_produced_, μg) was obtained using the following equation:

(1) $${DNA}_{produced}\;={\;O}_{content}*\frac{at{\%}_{execss}}{100}*\frac{100}{at{\%}_{label}}*\frac{100}{31.21}$$

where *DNA_produced_* represents the total amount of DNA produced (μg g^−1^ dry soil) during the 24 h incubation period. *O_content_* represents the total O content (μg) of the dried DNA extract. *at%_execss_* represents the at% excess ^18^O of the labeled sample minus the mean at% ^18^O of unlabeled samples. *at%_label_* represents the soil final water atom% ^18^O adjusted to 20 at the beginning. The constant of 31.21 represents the average weight% of O in DNA.

The conversion factor (CF) between microbial biomass carbon (MBC, μg g^−1^ DW) and soil DNA content (DNA_concentration_, μg g^−1^ DW) was obtained using the following equation:

(2) $$CF=\frac{MBC}{{DNA}_{concentration}}$$

The microbial growth rate based on dry soil (ng C g^−1^ h^−1^) was obtained using the following equation:

(3) $$C_{growth}=\frac{CF*{DNA}_{produced}*1000}{DW*t}$$

where *DW* represents the dry mass of soil (g), and *t* represents the incubation time in hours.

The microbial respiration flux (ng C g^−1^ h^−1^) was calculated based on the amount of CO_2_-C produced during the 24 h incubation period, as follows:

(4) $$C_{respiration}=\frac{(C_{treated}-C_{control})*M_{C}*V*298}{V_{m}*DW*273*t}$$

where *C_treated_* and *C_control_* represent the CO_2_ concentrations (ppm) measured in the treated and control samples, respectively, after the 24 h incubation period. *M_C_* represents the molecular mass of the element C (12 g mol^−1^); *V* represents the volume of the headspace in a vial (mL); *V_m_* represents the standard molar volume of ideal gas (L mol^−1^); *DW* represents the dry mass of soil (g); 273 and 298 represent the absolute temperature (K) at standard atmospheric pressure and the incubation temperature (K), respectively; and *t* is the incubation time in hours.

Finally, the microbial *CUE* was calculated according to the following equation:

(5) $$CUE=\frac{C_{gowth}}{C_{gowth}+C_{respiration}}$$

### 2.5. Microbial Community Structure and Diversity

Accurate 16S/ITS absolute quantification sequencing was conducted by Baihui Biotechnology Co., Ltd., Chengdu, China. Total genomic DNA samples were extracted using the MagBeads FastDNA Kit for Soil (MP Biomedicals, Irvine, CA, USA), according to the manufacturer’s instructions. The extracted DNA samples were stored at −20 °C prior to further analysis. The quantity and quality of the extracted DNA were measured using a NanoDrop NC2000 spectrophotometer (Thermo Fisher Scientific, Waltham, MA, USA) and agarose gel electrophoresis, respectively. PCR amplification was performed in the V4-V5 hypervariable region using primers 338F (5′-ACTCCTACGGGAGGCAGCAG-3′) and 806R (5′-GGACTACHVGGGTWTCTAAT-3′) for 16S rDNA in bacteria and primers ITS5F (5′-GGAAGTAAAAGTCGTAACAAGG-3′) and ITS1R (5′-GCTGCGTTCTTCATCGATGC-3′) for ITS in fungi. PCR amplicons were purified with Agencourt AMPure Beads (Beckman Coulter, Indianapolis, IN, USA) and quantified using the PicoGreen dsDNA Assay Kit (Invitrogen, Carlsbad, CA, USA). After the individual quantification step, amplicons were pooled in equal amounts, and paired-end sequencing (2 × 300 bp) was performed using the Illumina MiSeq platform with the MiSeq Reagent Kit v3.

The raw sequence was processed using QIIME2. The adapter and primer sequences were removed using the Cutadapt plugin [36], and then quality filtering and the identification of amplicon sequence variants (ASVs) were performed using the DADA2 plugin [37]. The classification and identification of representative ASV sequences used a pre-trained naive Bayes classifier for specific regions, with corresponding confidence thresholds set; a classifier trained on the RDP database (v11.5) was applied to 16S rRNA gene sequences (threshold = 0.8), and a classifier trained on the UNITE database (v9.0) was used for ITS2 sequences (threshold = 0.6). Then, the inserted sequences were identified and the number of reads was counted. Standard curves were generated for each sample based on the relationship between the number of reads and the number of inserted copies, and the absolute copy number of each ASV in each sample was calculated using the number of reads of the corresponding ASV. Since the inserted sequences did not belong to the microbial community of the sample, they needed to be removed in the subsequent analysis.

### 2.6. Data Statistics

One-way analysis of variance (ANOVA) was performed using IBM SPSS 22.0 to assess the effects of different salinity levels on microbial growth, respiration, CUE, POC, MAOC, and the soil microbial community structure and diversity. Correlation analysis, linear regression, and principal component analysis (PCA) were conducted to explore the relationships among these environmental variables. Furthermore, random forest modeling (RFM) and partial least squares path modeling (PLS-PM) were performed using the “vegan” and “plspm” packages in R to further elucidate the contributions and influence pathways of these factors regarding SOC in saline–alkali soil. All charts were generated using the Origin 2023 software, and all data presented in this study are expressed on a dry weight basis.

## 3. Results

### 3.1. Microbial C Turnover

**Figure 2:**
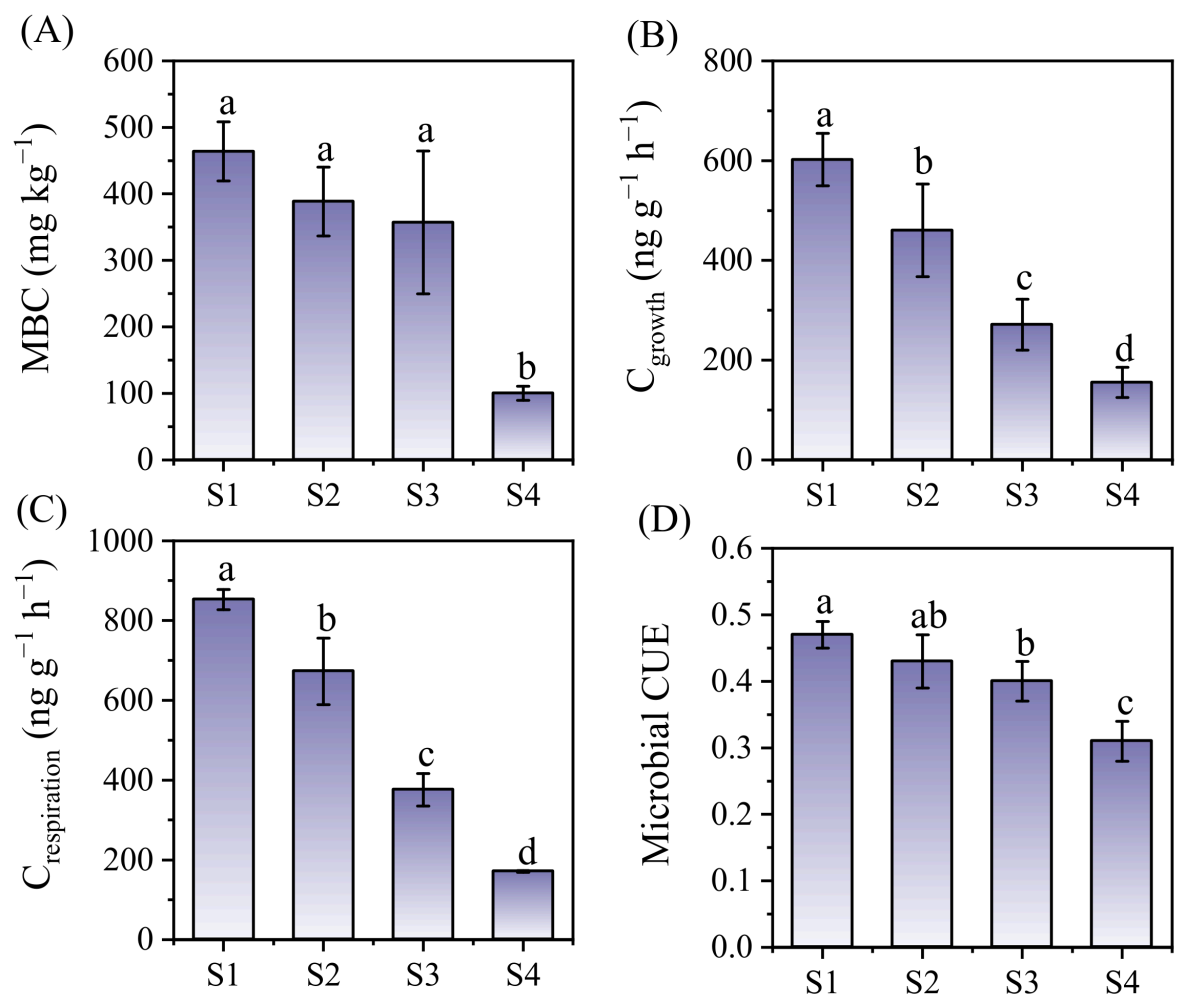
Microbial biomass carbon (MBC, (**A**)), microbial growth rate (C_growth_, (**B**)), respiration rate (C_respiration_, (**C**)), and carbon utilization efficiency (CUE, (**D**)) in soil at different sampling sites. S1, S2, S3 and S4 indicate different sampling sites. Data are expressed as means ± SD, and different letters indicate significance at the *p* < 0.05 level.

**Figure 3:**
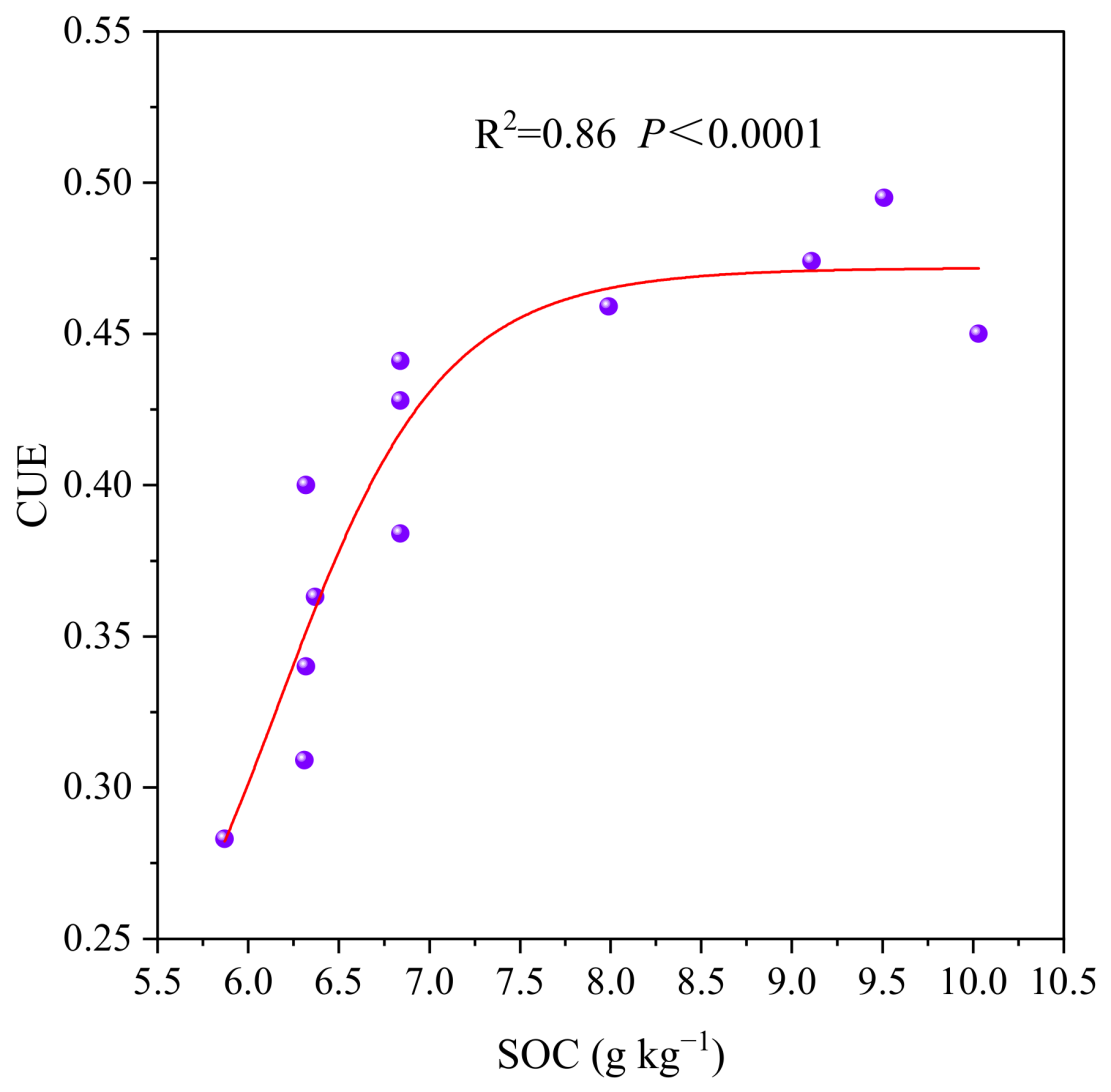
Relationship between microbial carbon utilization efficiency (CUE) and soil organic carbon (SOC) in saline–alkali soil.

The MBC, C_growth_, C_respiration_, and CUE of saline–alkali soil showed significant differences at the four sampling sites, as shown in Figure 2. The MBC at S4 was significantly lower than those at the other three sites, decreasing by 72.0% to 77.8%. Pairwise comparisons revealed a consistent order for both C_growth_ and C_respiration_, namely S1 > S2 > S3 > S4, and they showed significant differences between each pair of sites, indicating a marked decline in microbial growth and respiration with increasing salinity. The soil microbial CUE ranged from 0.31 to 0.47 across the four sites. Compared to S1, the CUE at S3 and S4 was significantly reduced by 14.9% and 34.0%, respectively, with that at S4 also being significantly lower than at S3. This result also indicates that microbial CUE shows a significant downward trend as the salinity level increases. As the SOC content increased, CUE followed a logarithmic growth pattern, stabilizing near its maximum (approximately 0.47) when SOC reached approximately 8 g kg^−1^ in saline–alkali soil (Figure 3). Both C_growth_ and C_respiration_ were positively and linearly correlated with CUE (R^2^ = 0.62, *p* ˂ 0.01; R^2^ = 0.78, *p* ˂ 0.0001, respectively) (Figure S1). In contrast, soil BD, pH, Ec, and Eh all showed significant negative linear correlations with CUE (R^2^ = 0.61–0.67, *p* ˂ 0.01) (Figure S2). Similarly, the concentrations of soluble Na^+^, Mg^2+^, Ca^2+^, Cl^−^, and SO_4_^2−^ and the total soluble salts were negatively correlated with CUE (R^2^ = 0.63–0.81, *p* ˂ 0.01), further confirming that CUE decreases with increasing soil salt content (Figure S3). A significant positive linear correlation was found between CUE and MBC, whereas no significant relationship existed between CUE and the SOC/TN ratio (Figure S4).

### 3.2. SOC Fractions

**Figure 4:**
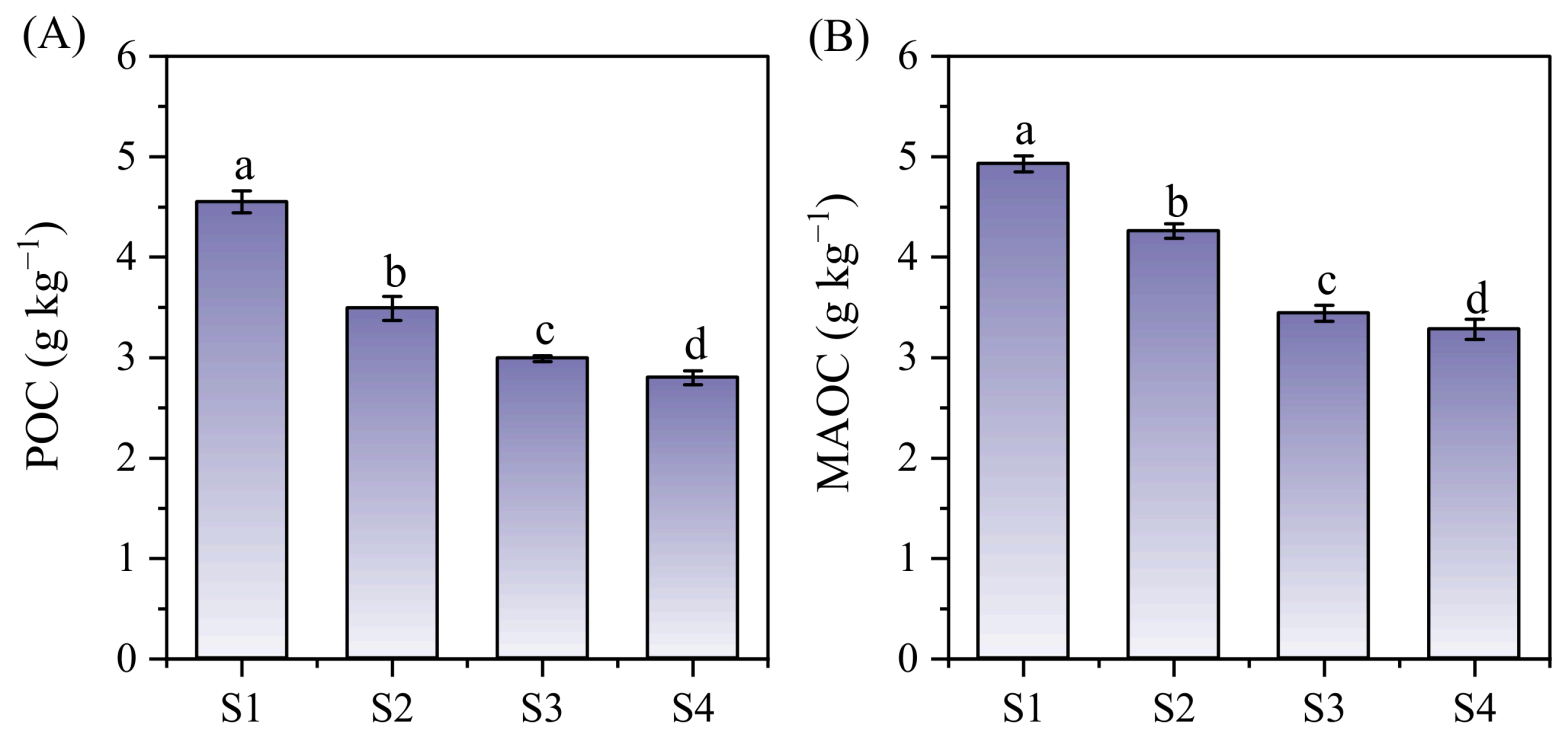
Content of particulate organic carbon (POC, (**A**)) and mineral-associated organic carbon (MAOC, (**B**)) in saline–alkali soil at different sampling sites. S1, S2, S3 and S4 indicate different sampling sites. Data are expressed as means ± SD, and different letters indicate significance at the *p* < 0.05 level.

**Figure 5:**
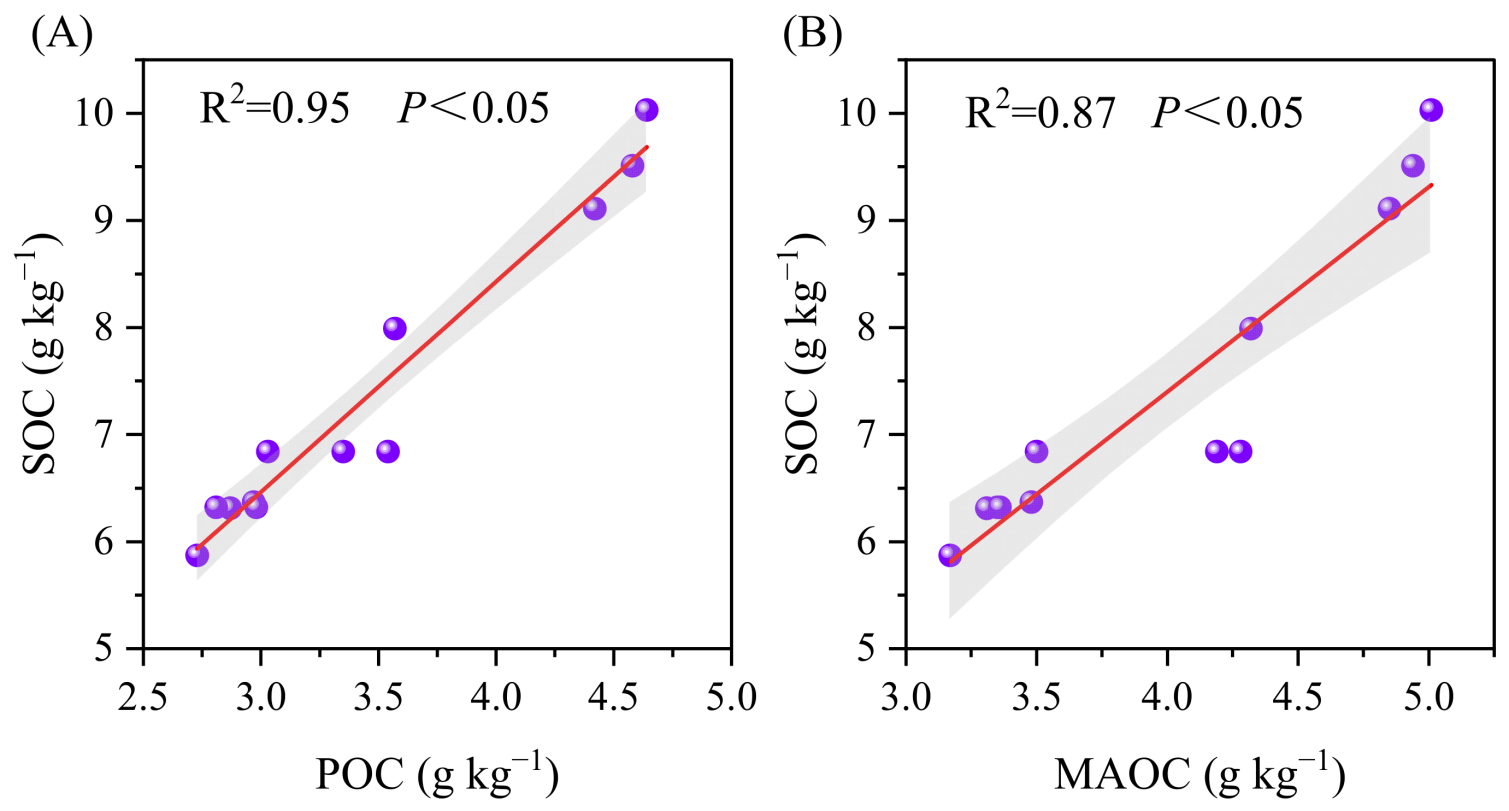
Relationships between soil organic carbon (SOC) and particulate organic carbon (POC, (**A**)) and mineral-associated organic carbon (MAOC, (**B**)) in saline–alkali soil.

The content of particulate organic carbon (POC) and mineral-associated organic carbon (MAOC) in the saline–alkali soil from the four different sampling sites is presented in Figure 4. Both fractions exhibited a consistent trend across sites, with significant pairwise differences following the order S1 > S2 > S3 > S4. At all sites, the proportion of MAOC in SOC (52.04–55.02%) was significantly higher than that of POC (44.98–47.96%). POC and MAOC each showed a significant positive linear correlation with SOC (R^2^ = 0.95, *p* ˂ 0.05; R^2^ = 0.95, *p* ˂ 0.05) (Figure 5), indicating that the SOC content is closely related to these carbon fractions and increases with their accumulation in saline–alkali soil. Furthermore, both POC and MAOC were significantly and positively correlated with microbial CUE, suggesting that higher content of these organic carbon fractions is associated with increased CUE (Figure S4).

### 3.3. Soil Bacterial and Fungal Communities

**Figure 6:**
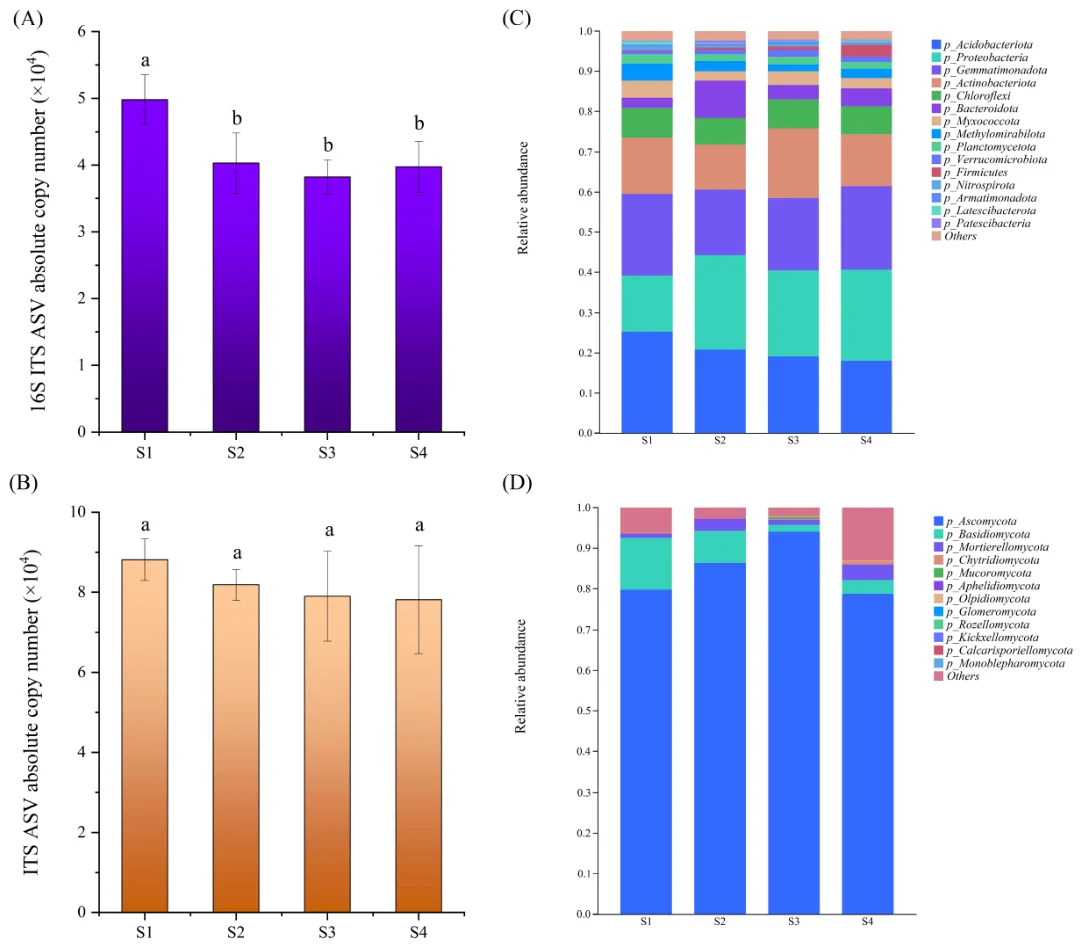
Change features of soil microbial community diversity and composition. Absolute abundances (copy numbers) (**A**,**B**) and relative abundance at the phylum level (**C**,**D**) of bacterial and fungal communities in soil at different sampling sites. S1, S2, S3 and S4 indicate different sampling sites. Data in (**A**,**B**) are expressed as means ± SD, and different letters indicate significance at the *p* < 0.05 level.

The alpha diversity of the soil bacterial and fungal communities is presented in Table 2. With increasing salinity, no significant change was observed in the bacterial Chao1 index. The bacterial Observed index at S1 was significantly lower than at the other three sites. The bacterial Shannon index at S3 was significantly higher than at S1 and S2, but it showed no significant difference compared to S4. Regarding fungal communities, the Chao1 index at S1 was significantly lower than at the other three sites, whereas no significant difference in the Shannon index was detected among the four sites. The absolute copy number of the 16S rRNA gene at S1 was significantly higher than those at the other three sites, with reductions at S2, S3, and S4 of 19.0%, 28.8%, and 26.4%, respectively (Figure 6A). In contrast, no significant differences in ITS gene copy numbers were observed across the four sites (Figure 6B).

**Figure 7:**
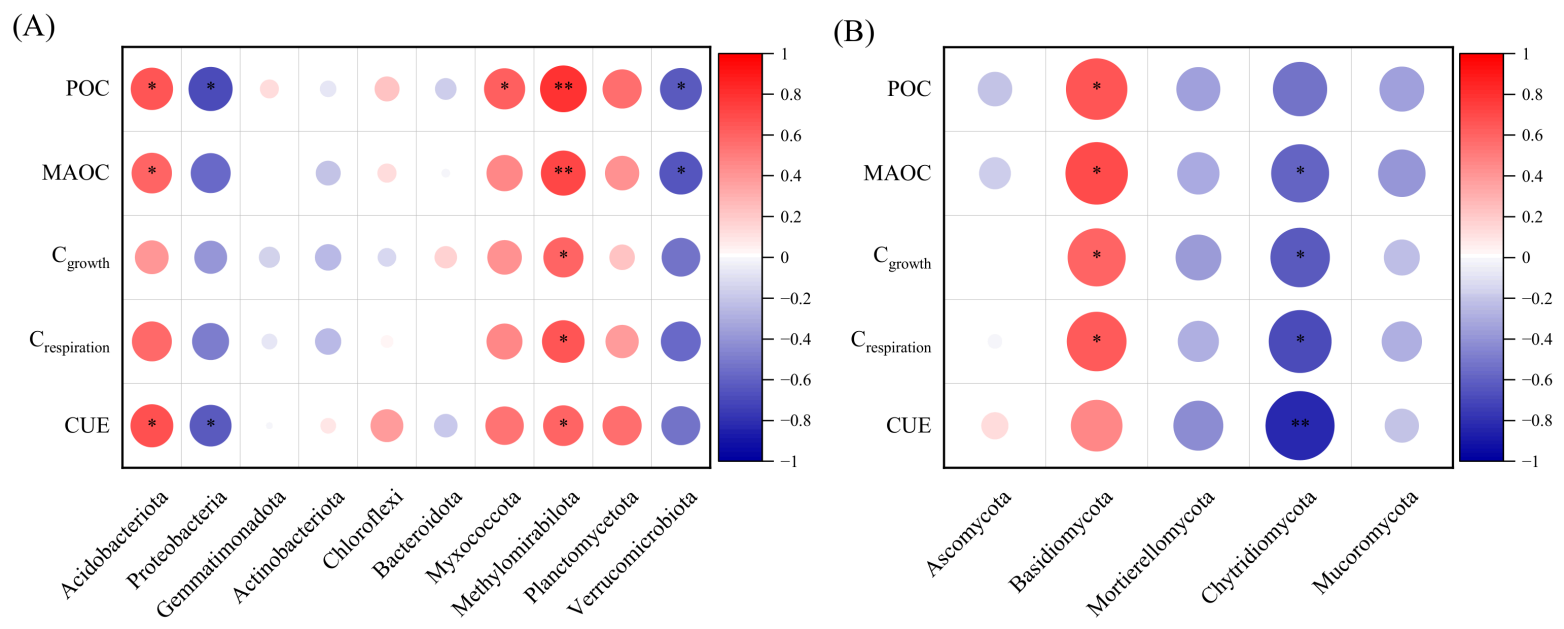
Correlations between soil carbon turnover and bacterial (**A**) and fungal communities (**B**); * and ** indicate significant differences at the *p* < 0.05 and *p* < 0.01 levels, respectively. The circle size indicate the magnitude of the correlation coefficient.

At the phylum level, the soil bacterial communities were predominantly composed of *Acidobacteriota*, *Proteobacteria*, *Gemmatimonadota*, *Actinobacteriota*, and *Chloroflexi*, each with a relative abundance exceeding 5% (Figure 6C). From S1 to S4, as the salinity increased, the relative abundances of *Acidobacteriota*, *Gemmatimonadota*, and *Chloroflexi* showed a gradually decreasing trend, whereas those of *Proteobacteria* and *Actinobacteriota* showed an increasing trend. Fungal communities were overwhelmingly dominated by *Ascomycota*, with relative abundances ranging from 78.8% to 94.1%, followed by *Basidiomycota*, *Mortierellomycota*, *Chytridiomycota*, and *Mucoromycota*, which together accounted for only 3.53–13.8% (Figure 6D). The remaining fungal phyla occurred at low abundances and only at some sites. From S1 to S4, the relative abundance of *Ascomycota* first increased and then decreased, peaking at S3, suggesting that higher salinity levels may inhibit the activity of this dominant fungal phylum. *Basidiomycota* showed a gradual decline from S1 to S4, whereas *Mortierellomycota* exhibited an increasing trend. Furthermore, correlation analyses between microbial taxa and carbon cycling revealed that bacterial *Acidobacteriota*, *Proteobacteria*, *Methylomirabilota*, and *Verrucomicrobiota* and fungal *Basidiomycota* and *Chytridiomycota* play crucial roles in carbon cycling (Figure 7A,B).

### 3.4. Potential Drivers of SOC Turnover in Saline–Alkali Soil

**Figure 8:**
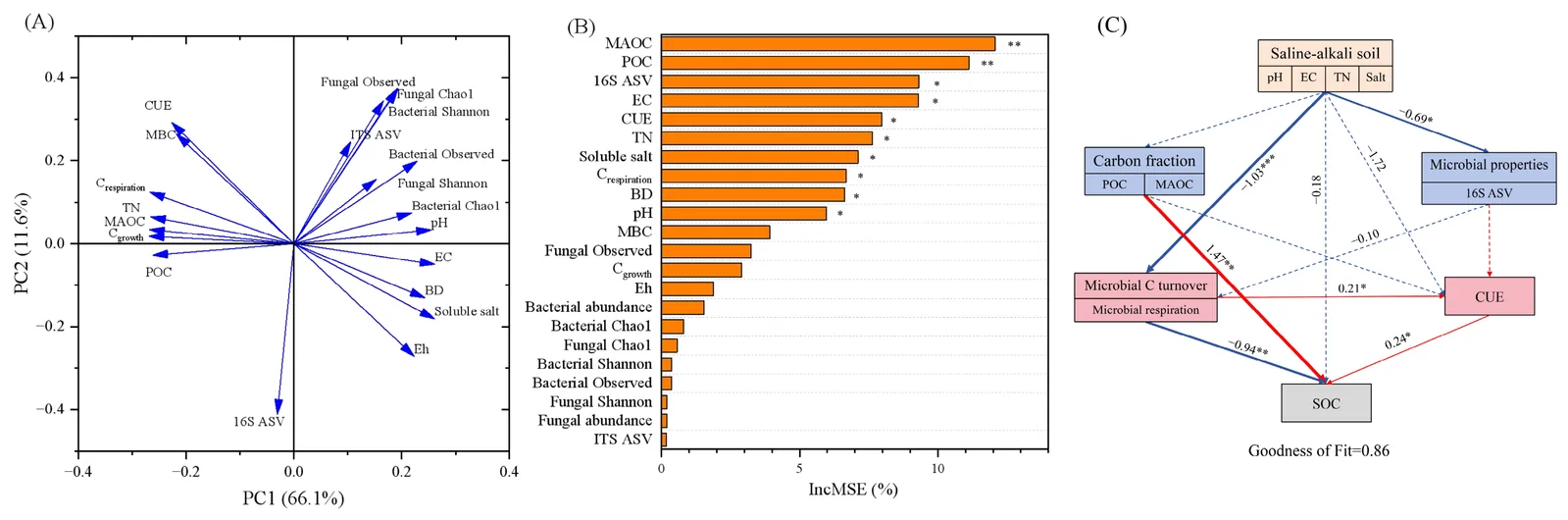
PCA analysis among environmental factors (**A**) and the relative importance of soil properties, carbon fractions, microbial C turnover, and microbial properties for soil organic carbon (SOC) in saline–alkali soils (**B**) based on a random forest model (RFM). Partial least squares path modeling (PLS-PM) showed the effects of soil properties, carbon fractions, microbial C turnover, and microbial properties on soil organic carbon (SOC) in saline–alkali soils (**C**). *, ** and *** indicate significant effects at the *p* < 0.05, *p* < 0.01 and *p* < 0.001 levels, respectively.

Principal component analysis (PCA) indicated that the first two principal components (PC1) accounted for 77.7% of the total variance. Key variables including the pH, POC, MAOC, C_growth_, C_respiration_, bacterial Chao1, and fungal Shannon index were strongly associated with PC1 and primarily linked to soil salinity and alkalinity levels (Figure 8A). Random forest modeling (RFM) identified the carbon fraction, bacterial community structure, CUE, respiration, and soil salinization indicators as the main drivers of SOC (Figure 8B). Partial least squares path modeling (PLS-PM, GOF = 0.86) further indicated that the saline–alkali soil variables strongly affected the absolute copy number of the 16S rRNA gene and microbial carbon respiration (Figure 8C). Carbon fractions had a positive impact on SOC, and microbial carbon respiration positively influenced CUE but negatively impacted SOC, whereas CUE showed a positive effect on SOC. These results also confirm that the poor properties of saline–alkali soil lead to a decrease in organic carbon fractions and microbial respiration, which directly or indirectly causes lower microbial CUE, resulting in low SOC content or carbon sequestration capacity. Moreover, the path coefficient also revealed that organic carbon fractions and microbial respiration, rather than CUE, are crucial drivers of SOC in saline–alkali soils.

## 4. Discussion

### 4.1. Effects of Saline–Alkali Stress on Soil Microbial Carbon Turnover

Our results revealed that microbial CUE gradually decreased along the salinity gradient from 0.47 at S1 to 0.31 at S4, accompanied by corresponding reductions in carbon growth and respiration (Figure 2). At the highest salinity site, S4, microbial biomass carbon (MBC) was reduced by 72.0–77.8% compared to other sites. Consistent with these findings, we observed significant negative linear correlations between CUE and key salinity indicators including soil BD, pH, Ec, Eh, soluble Na^+^, Mg^2+^, Ca^2+^, Cl^−^, and SO_4_^2−^, and total soluble salt ions (Figures S2 and S3), confirming that increasing salinity directly inhibits microbial carbon metabolism. Soil salinity and alkalinity reduce microbial CUE through multiple interrelated physiological and ecological mechanisms. Increased soluble salt concentrations in soil raise the osmotic pressure outside microbial cells, inducing ion toxicity and disrupting intracellular homeostasis. Under such conditions, microorganisms must transfer a large amount of carbon and energy from biosynthesis to osmotic regulation to maintain the osmotic pressure and ionic balance of the cytoplasm [38]. This highly energy-consuming adaptation mechanism directly reduces the proportion of absorbed carbon used for growth and biomass formation, thereby reducing CUE [39]. Furthermore, PLS-PM analysis confirmed this explanation: the salinity variable significantly affected microbial respiration and the 16S rRNA gene abundance (Figure 8C), indicating that the microbial metabolic capacity was inhibited under stress conditions. Microbial communities in saline–alkali soils typically face energy limitations, which inevitably reduces the carbon conversion efficiency [40].

We observed a logarithmic increase in CUE with increasing SOC content, which stabilized when SOC reached approximately 8 g kg^−1^ (Figure 3). This finding further suggests that, although moderate SOC levels enhance CUE, excessive salinity stress reduces carbon availability, becoming the primary limiting factor. Previous studies have shown that microbial biomass and enzyme activity in arid soils sharply decline when the electrical conductivity (EC) approaches 4 dS m^−1^, possibly due to more microbes entering non-active or dormant states in highly saline soils [17]. Similarly, one study found that the microbial biomass and respiration rates were lower in hypersaline wetlands than in freshwater wetlands, despite higher dissolved organic carbon (DOC) levels, indicating the suppression of microbial colonization and carbon transformation [41]. The carbon supply index (DOC/MBC) reached its maximum in areas with the lowest microbial activity, suggesting that, under extreme salinity conditions, the limitation on microbial metabolism lies not in carbon availability but in the microbes’ ability to acquire and utilize carbon. Additionally, our results showed that both C_growth_ and C_respiration_ were positively correlated with CUE, emphasizing that microbial growth and respiration in saline–alkaline soils are tightly coupled processes rather than independent ones. Under saline–alkaline conditions, reduced microbial growth rates lead to decreased biomass turnover, thereby limiting the production of microbial necromass, the primary precursor for stable SOC formation [42]. A global meta-analysis revealed that salinization reduces soil organic carbon by 21% and microbial necromass carbon contributes 32–39% less to SOC in saline–alkaline soils than in non-saline soils [43]. Collectively, these findings demonstrate that salinity stress primarily suppresses microbial CUE by increasing energy expenditure for osmoregulation, shifting microbial communities toward dormancy or inactivity, and inhibiting synthetic metabolic processes driven by respiration.

### 4.2. Effects of Saline–Alkali Stress on Soil Organic Carbon Fractions

POC is primarily derived from coarse plant debris and remains loosely associated with sand-sized particles, whereas MAOC is stabilized through chemical binding and sorption onto clay minerals and metal oxides, making it the more persistent form of SOC in most ecosystems [44]. Our data demonstrated consistent declines in both POC and MAOC across the four sites, with MAOC consistently accounting for the majority of SOC (52.04–55.02%) compared to POC (44.98–47.96%) at all sites (Figure 4). Both fractions were significantly positively correlated with SOC and CUE (Figure S4), indicating that the reduction in organic carbon fractions caused by salinity was closely related to the decline in microbial carbon conversion efficiency. In saline–alkali soils, MAOC is largely formed from low-molecular-weight microbial metabolites and necromass that can access mineral surfaces. In contrast, POC accumulation depends more directly on plant residue inputs and physical protection within macro-aggregates. Our results showed a consistent MAOC/POC ratio across all sites (approximately 1.1:1), indicating that the relative partitioning between fractions is relatively insensitive to salinity within the range studied, but the absolute carbon content in both pools declines proportionally as salt stress intensifies. There was a strong positive correlation between POC and MAOC and microbial CUE (Figure 5), indicating the ability of the microbial community to convert assimilated carbon into stable forms efficiently, directly influencing the accumulation of these two organic carbon fractions. However, the mechanisms linking CUE to MAOC and POC may differ. For MAOC, which relies on microorganisms to convert plant residues into low-molecular-weight compounds and adsorb them onto mineral surfaces, a decrease in CUE under high-salinity conditions would inhibit the generation of microbial metabolites and residue substances, which are precursors for MAOC formation [17]. In contrast, the accumulation of POC may be more directly related to the input of plant residues and the physical protection provided by aggregates, both of which are indirectly affected by salinity, which reduces plant productivity and destabilizes aggregate stability [45]. A study covering saline–alkali areas in China found that the accumulation of plant-derived carbon in all salinized areas significantly increased, and the ratio of plant-derived carbon to microbial-derived carbon also increased accordingly [2]. These results indicate that, after the alleviation of salt stress, the soil can restore its carbon sequestration capacity, with plant-derived carbon mainly contributing to POC and microbial-derived carbon mainly contributing to MAOC.

Our research results indicate that the decrease in CUE (from 0.47 to 0.31) caused by high salinity is likely to directly reduce microbial necromass C, thereby limiting the accumulation of MAOC. This is because bacterial and fungal necromass are the main substrates for the stability of mineral-associated organic carbon. The critical role of microbial necromass formation in SOC sequestration in saline soils is further substantiated by recent studies. Green manure root return in saline–alkali cropping systems was shown to increase microbial necromass C, particularly bacterial necromass C, by 1.6–2.8 times, and this was the primary driver of SOC sequestration [46]. Moreover, the subsurface application of organic amendments in saline soils increased microbial necromass accumulation in mineral-associated organic matter, and increased microbial CUE was a key factor driving this enhancement [47]. Conversely, a study on straw decomposition under salinity revealed that high soil salinity decreased microbial biomass and diversity, slowed down straw decomposition, lowered microbial necromass by 13%, and increased plant-derived C by 6.9% compared to low soil salinity, indicating that, under salt stress, the microbial contribution to SOC is replaced by less efficiently stabilized plant-derived carbon [3]. Thus, the inhibition of microbial metabolism caused by salinization reduces MAOC production and causes carbon to exist in a more unstable form, seriously weakening the long-term carbon sequestration capacity of the soil.

### 4.3. Microbial Communities and Their Influence on SOC Sequestration

Bacterial alpha diversity exhibited differential responses to increasing salinity; the Chao1 index remained stable, while the Observed index at S1 was significantly lower than at other sites; and the Shannon index at S3 was significantly higher than at S1 and S2 (Table 2). The S1 site may have been frequently subjected to human interference, resulting in a relatively low Observed index. However, under the long-term influence of salinity, the S3 site may represent a transitional ecological niche, where some local species that have adapted to low salinity are retained and moderately salt-tolerant species are also accommodated, thus presenting the highest diversity [48]. The fungal Chao1 at S1 was significantly lower than at other sites, with no significant differences in Shannon across sites. Previous studies have also shown that salinity exerts strong selective pressure on microbial communities by increasing osmotic stress and ionic toxicity, thereby reducing overall microbial diversity and facilitating the survival of soil salt-tolerant communities [9]. However, the fungal Shannon diversity in this study did not show a significant decline, which indicates that fungal communities exposed to long-term saline–alkali environments may have buffered the negative impact of increasing salinity on diversity through species selection and functional redundancy.

At the phylum level, the increasing salinity from S1 to S4 reduced the relative abundances of bacterial *Acidobacteriota*, *Gemmatimonadota*, and *Chloroflexi*, while increasing those of *Proteobacteria* and *Actinobacteriota* (Figure 6C). Correlation analyses identified *Acidobacteriota*, *Proteobacteria*, *Methylomirabilota*, *Verrucomicrobiota*, *Basidiomycota*, and *Chytridiomycota* as taxa playing crucial roles in carbon cycling (Figure 7A). A recent study also found that the relative abundance of *Proteobacteria* increased from 20.0% under non-saline conditions to 38.0% under highly saline conditions, suggesting that it may act as an indicator microorganism for salinity [49]. Similarly, the *Actinobacteria* phylum in high-alkalinity and -salinity soil was confirmed to be significantly enriched in previous studies [50]. These two phyla have extensive metabolic capabilities, containing many salt-tolerant bacterial genera; they can maintain carbon metabolism under osmotic stress and mainly maintain intracellular ion homeostasis by producing compatible solutes and expressing high-affinity potassium absorption systems. However, the carbon cost of osmotic adjustment forces microbes to prioritize survival over growth, directly lowering CUE. In contrast, the decline of *Acidobacteriota*, *Gemmatimonadota*, and *Chloroflexi* reflects their generally greater sensitivity to salinity, which may be due to their more specialized metabolic niches and the absence of robust salt acclimation mechanisms [51]. The differences in the responses of the *Proteobacteria* and *Acidobacteriota* phyla to salinity have significant implications for the carbon cycle in saline–alkali soils. According to our research results, *Proteobacteria* are identified as an important group that plays a key role in the carbon cycle (Figure 7A,B). However, the enrichment of the *Proteobacteria* phylum under saline–alkali conditions does not necessarily enhance the overall carbon fixation capacity, because the specific functional characteristics of the dominant *Proteobacteria* phylum lineages change with increasing salinity. A study on saline–alkali soil indicated that, under high-salinity conditions, stress-tolerant S-strategists dominate, resulting in the lowest accumulation of microbial necromass; after 1000 years of cultivation, as the salinity decreases and nutrient availability increases, the microbial community gradually shifts toward high-yield Y-strategists, which represent the greatest contribution of microbial necromass to the SOC pool [52]. This temporal variation provides strong evidence supporting our research results. Under high-salt conditions (S3, S4), the dominant *Proteobacteria* phylum is likely to provide S-type strategy bacteria, which prioritize survival rather than the accumulation of biomass and residues. In contrast, the *Proteobacteria* phylum may contain more Y-type strategy bacteria in low-salt soil, which can efficiently convert carbon into stable residues. Although this study has achieved some progress in understanding the carbon sequestration functions of microorganisms, the microbial predictive functional analysis merely indicates the metabolic potential implied by the community composition. Therefore, the carbon sequestration mechanism in saline–alkali soils still requires further verification through metagenomic transcriptomics or isotope tracing.

Fungal communities were overwhelmingly dominated by *Ascomycota* (78.8–94.1%), with the *Ascomycota* abundance peaking at S3, *Basidiomycota* declining gradually, and *Mortierellomycota* increasing along the salinity gradient (Figure 6D). Correlation analyses identified *Basidiomycota* and *Chytridiomycota* as taxa playing crucial roles in carbon cycling (Figure 7B). This result is in line with the widely accepted consensus, as the *Ascomycota* phylum contains a large number of saprophytic and stress-tolerant species, which can adapt to various extreme environmental conditions, including high salinity, low water potential, and high pH [53]. The abundance of the *Ascomycota* phylum reached its peak at the S3 stage and then decreased at the S4 stage (Figure 6), which indicates that moderate salinization may increase the quantity and activity of these fungi by reducing their competition with other groups, while extreme salinization would bring cumulative metabolic costs and ultimately inhibit these highly tolerant fungal strains. The *Ascomycota* phylum, which includes many ectomycorrhizal and wood-decomposing fungi, significantly contributes to the recalcitrant organic carbon pool. This phylum decreased gradually from S1 to S4, which is consistent with its generally lower salt tolerance. Notably, as the salinity increases, the abundance of *Basidiomycota* rises, as it contains species that can produce trehalose and other osmotic protective substances. This indicates that these fungi may have unique adaptive characteristics that enable them to grow well in high-salt environments and help to enhance stress resistance in the carbon cycle [49]. Through the relative dominance of *Ascomycota* and the decrease in the number of *Basidiomycota*, we indirectly observed that the shift from a fungal-dominated to a bacterial-dominated microbial residue may have weakened SOC’s physical protection, as fungal hyphae are more effective than bacteria in stabilizing aggregates. Therefore, the SOC formed in saline soil is not only smaller in quantity but also less stable. Furthermore, we found that the absolute copy number of the 16S rRNA gene decreased significantly by 19.0%, 28.8%, and 26.4% at S2, S3, and S4, respectively, compared to S1, while there was no significant difference in the ITS copy number among the various sites (Figure 6A,B). A previous study also revealed that the bacterial diversity declined sharply along a salinity gradient, while the fungal Shannon diversity remained stable [54]. Critically, this study shows that salinity indirectly reduced soil multifunctionality primarily through bacterial pathways, while fungal contributions were marginal. The decline in bacterial abundance directly explains the reduced metabolic capacity for carbon transformation and the associated decreases in CUE and MAOC formation observed in our study. This asymmetric response at moderate salinity suggests stronger nutrient limitations on bacteria than fungi, as bacteria lack the filamentous networks and osmotic regulation that sustain fungal metabolism under salt stress. Consequently, tolerant taxa maintained basal carbon cycling but produced low biomass and residues (reflected by reduced MBC and CUE), limiting microbial contributions to stable organic carbon pools. Increased salinity exacerbated limitations in carbon and nitrogen, prompting a shift in the life history strategy from the Y-type to the S-type strategy and driving the community structure in a more deterministic environmental selection direction. These factors collectively restricted the biomass turnover efficiency and the formation of SOC produced by microorganisms. Overall, the observed changes in community structure resulted from the combined effects of long-term selection and salinity gradients. The local microbial community may have buffered some of the decline in diversity, but it failed to reverse the trend of reduced carbon metabolic efficiency. This provides a more realistic ecological perspective for understanding the limitations of carbon sequestration in naturally saline–alkali soils.

### 4.4. Microbial Mechanisms for SOC Storage in Saline–Alkali Soils

The RFM and PLS-PM results also confirm that the poor properties of saline–alkali soil lead to a decrease in organic carbon fractions and microbial respiration (Figure 8B,C), which directly or indirectly causes lower microbial CUE, resulting in the characteristic of low SOC content or carbon sequestration capacity in saline–alkali soil. A previous study also found that an increase in salt concentration significantly inhibited the organic carbon used for respiration in the soil, and this phenomenon has been consistently observed in both short-term and long-term salt-affected conditions [9]. Moreover, organic carbon fractions and microbial respiration, rather than CUE, are the direct drivers of SOC in saline–alkali soils. Some studies have also shown that microbial respiration may better reflect the dynamics of SOC than CUE in certain ecosystems [14,55]. Overall, organic carbon fractions and microbial respiration, rather than CUE, act as the direct drivers of soil organic carbon (SOC) dynamics in saline–alkali soils; salinization limited microbial growth and respiration, reduced CUE, and restructured microbial communities toward stress-adapted yet metabolically less efficient taxa. These microbial constraints, in turn, lowered the accumulation of organic carbon fractions and the total SOC pool. These findings demonstrate that saline–alkali stress restricts SOC sequestration primarily by weakening microbial activity and community function, with CUE playing only an indirect role. Nevertheless, the carbon sequestration mechanism elucidated in this study has a clear regional dependence. The research results provide a reference and insights for improving other saline–alkali soils with similar soil texture characteristics in regions with similar climatic conditions (such as semi-arid areas with intense evaporation). However, caution is needed when dealing with regions with significantly different environmental conditions. In regions with large climate differences and significant differences in soil properties, the relative weights of respiratory activity and microbial carbon utilization efficiency (CUE) in regulating soil organic carbon (SOC) may be altered. Additionally, the salinity threshold at which the microbial community structure undergoes a significant transformation may also vary due to differences in background nutrients and pH conditions. Therefore, although the core mechanism of this study has universal applicability, the results should be locally parameterized when directly applied to management practices in different regions. Future research will include cross-site comparisons along drought or salinity gradients to further test and enhance the applicability of the research results.

## 5. Conclusions

As the salt and alkali levels increased, the microbial CUE declined significantly from 0.47 to 0.31; correspondingly, both the microbial growth and respiration rates also showed a downward trend, and MBC decreased by 72.0–77.8% at S4. Both particulate organic carbon (POC) and mineral-associated organic carbon (MAOC) decreased with increasing salinity, with MAOC accounting for 52.04–55.02% of SOC across all sites. Strong positive correlations between POC, MAOC, and CUE indicated that reduced microbial carbon turnover efficiency directly limits stable carbon pool formation. Salinity shifted bacterial communities toward salt-tolerant *Proteobacteria* and *Actinobacteriota* while suppressing sensitive phyla such as *Acidobacteriota*, *Gemmatimonadota*, and *Chloroflexi*. Fungal communities remained dominated by *Ascomycota* (78.8–94.1%), with *Basidiomycota* declining and *Mortierellomycota* increasing along the salinity gradient. The absolute copy number of the 16S rRNA gene decreased by 19.0–28.8% under high salinity, whereas the ITS copy number showed no significant change. Partial least squares path modeling further revealed that organic carbon fractions and microbial respiration, rather than CUE, are the direct drivers of SOC in saline–alkali soils. In conclusion, these findings establish that saline–alkali stress reduces SOC sequestration primarily by inhibiting microbial growth and respiration, lowering CUE, and shifting microbial communities toward stress-adapted but less efficient carbon metabolizers. These changes ultimately limit the accumulation of organic carbon fractions and the total SOC pool. Moreover, in saline–alkali soils, organic carbon fractions and microbial respiration, rather than CUE, serve as the major drivers of SOC dynamics.

## Figures and Tables

**Table 1:** Main properties of soils at four different sampling sites.

Indicator	S1	S2	S3	S4
BD (g cm^−3^)	1.34 ± 0.02 b	1.38 ± 0.01 b	1.52 ± 0.04 a	1.53 ± 0.03 a
pH	7.98 ± 0.07 c	8.73 ± 0.01 b	8.75 ± 0.18 b	9.06 ± 0.11 a
Ec (μS cm^−1^)	342.67 ± 25.79 c	662.00 ± 7.21 b	1546.00 ± 42.44 a	1617.33 ± 63.96 a
Eh (mV)	183.53 ± 4.04 b	185.32 ± 7.09 b	190.63 ± 4.51 b	205.42 ± 1.15 a
Soluble Na^+^ (mg kg^−1^)	1335.25 ± 33.40 d	1438.26 ± 43.86 c	1794.38 ± 19.72 b	1987.61 ± 17.36 a
Soluble Ca^2+^ (mg kg^−1^)	183.33 ± 44.31 c	334.07 ± 28.81 b	400.37 ± 10.36 ab	469.93 ± 73.52 a
Soluble Mg^2+^ (mg kg^−1^)	97.68 ± 21.34 c	253.18 ± 35.08 b	263.30 ± 35.08 b	432.51 ± 47.64 a
Soluble Cl^−^ (mg kg^−1^)	231.87 ± 50.09 b	268.37 ± 36.14 b	303.80 ± 33.26 b	388.97 ± 36.31 a
Soluble SO_4_^2−^ (mg kg^−1^)	137.97 ± 2.11 b	204.22 ± 51.14 b	259.37 ± 28.44 b	371.61 ± 15.55 a
Total soluble salt (g kg^−1^)	1.99 ± 0.04 d	2.50 ± 0.04 c	3.02 ± 0.03 b	3.73 ± 0.03 a
SOC (g kg^−1^)	9.55 ± 0.47 a	7.23 ± 0.66 b	6.51 ± 0.29 bc	6.17 ± 0.26 c
TN (g kg^−1^)	0.96 ± 0.02 a	0.86 ± 0.02 b	0.77 ± 0.03 c	0.72 ± 0.04 c
SOC/TN	9.96 ± 0.73 a	8.42 ± 0.69 b	8.44 ± 0.24 b	8.53 ± 0.18 b

Note: Data are expressed as means ± SD, and different letters indicate significance at the *p* < 0.05 level.

**Table 2:** Alpha diversity indices (Chao1, Observed, and Shannon indices) of soil microbial communities at four sampling sites.

Sampling Site	Bacterial Community			Fungal Community		
	Chao1	Observed	Shannon	Chao1	Observed	Shannon
S1	3426.4 ± 179.3 a	3232.3 ± 151.7 b	10.40 ± 0.10 b	215.64 ± 34.38 b	214.3 ± 32.5 b	4.95 ± 0.43 a
S2	3511.5 ± 216.3 a	3414.7 ± 165.4 a	10.51 ± 0.19 b	294.61 ± 35.49 a	293.0 ± 33.4 a	5.52 ± 0.82 a
S3	3819.0 ± 415.4 a	3748.3 ± 374.1 a	10.85 ± 0.19 a	292.06 ± 39.9 a	291.3 ± 39.8 a	5.9 ± 0.19 a
S4	3966.1 ± 272.0 a	3706.7 ± 198.4 a	10.59 ± 0.15 ab	286.66 ± 33.52 a	285.0 ± 34.0 a	5.59 ± 0.43 a

Note: S1, S2, S3 and S4 indicate different sampling sites. Data are expressed as means ± SD, and different letters indicate significance at the *p* < 0.05 level.

## Data Availability

Data are available from the authors upon request.

## Supplementary Materials

The following supporting information can be downloaded at https://www.mdpi.com/article/10.3390/biology15161335/s1. Figure S1: Relationships of the microbial carbon use efficiency (CUE) with microbial growth rate (C_growth_, (A)), and microbial respriation rate (C_respriation_, (B)) in saline-alkali soil; Figure S2: Relationships of the microbial carbon use efficiency (CUE) with soil bulk density (BD, (A)), pH (B), electric conductivity (Ec, (C)), redox potential (Eh, (D)) in saline-alkali soil; Figure S3: Relationships of the microbial carbon use efficiency (CUE) with total soluble salt (A), soluble Na^+^ (B), soluble Mg^2+^ (C), soluble Ca^2+^ (D), soluble Cl^−^ (E), soluble SO_4_^2−^ (F) in saline-alkali soil; Figure S4: Relationships of the microbial carbon use efficiency (CUE) with particulate organic carbon (POC, (A)), mineral-associated organic carbon (MAOC, (B)), microbial biomass carbon (MBC, (C)), and SOC/TN (D) in saline-alkali soil.
