# Supplementary material for: Regulatory Mechanism of Carbon Fractions and Microbial Metabolism on Organic Carbon Turnover in Saline–Alkali Soils
Source: Biology (Basel). 2026 Aug 7;15(16):1335. doi: 10.3390/biology15161335 (PMC13509726; doi:10.3390/biology15161335)
Supplement: Supplementary file 1 [file biology-15-01335-s001.zip › biology-4455801-supplementary.pdf]

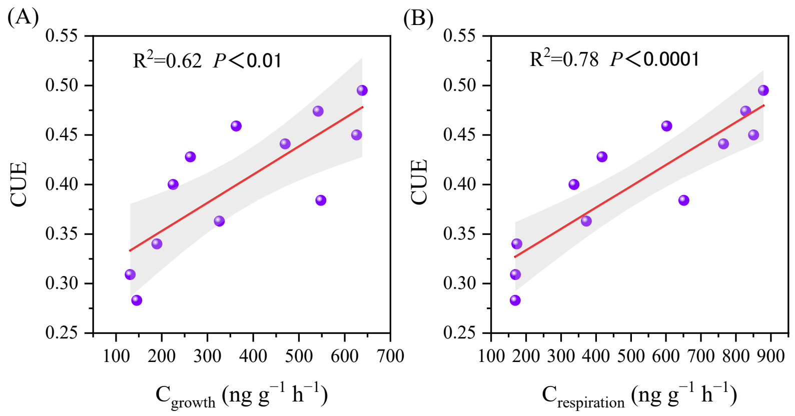

Figure S1. Relationships of the microbial carbon use efficiency (CUE) with microbial growth rate (C_{growth}, (A)), and microbial respriation rate ($C_{respriation}$, (B)) in saline–alkali soil.

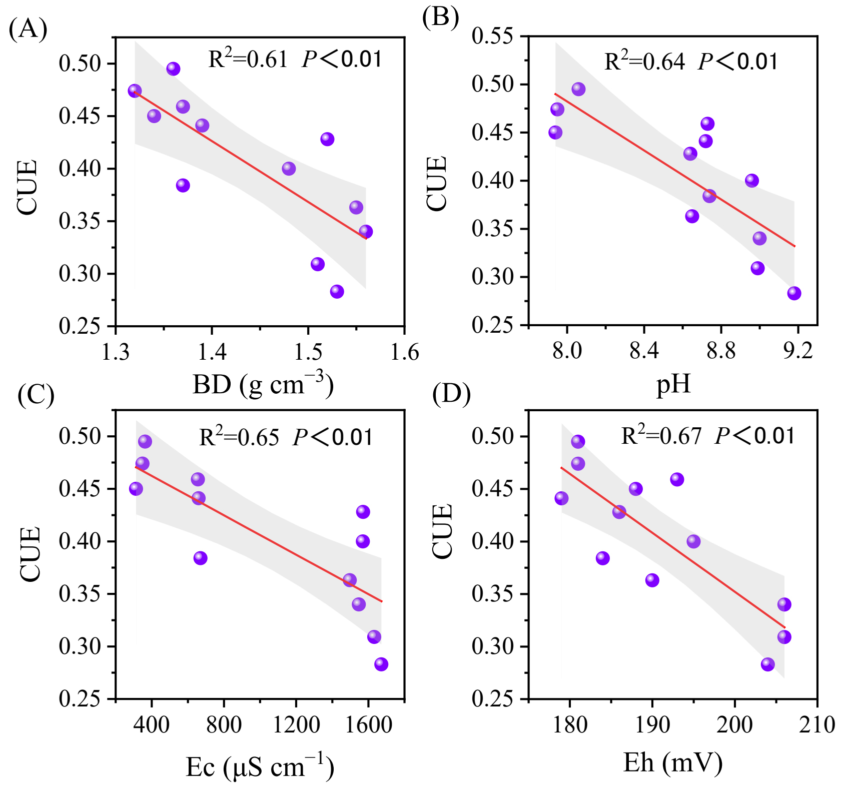

Figure S2. Relationships of the microbial carbon use efficiency (CUE) with soil bulk density (BD, (A)), pH (B), electric conductivity (Ec, (C)), redox potential (Eh, (D)) in saline–alkali soil.

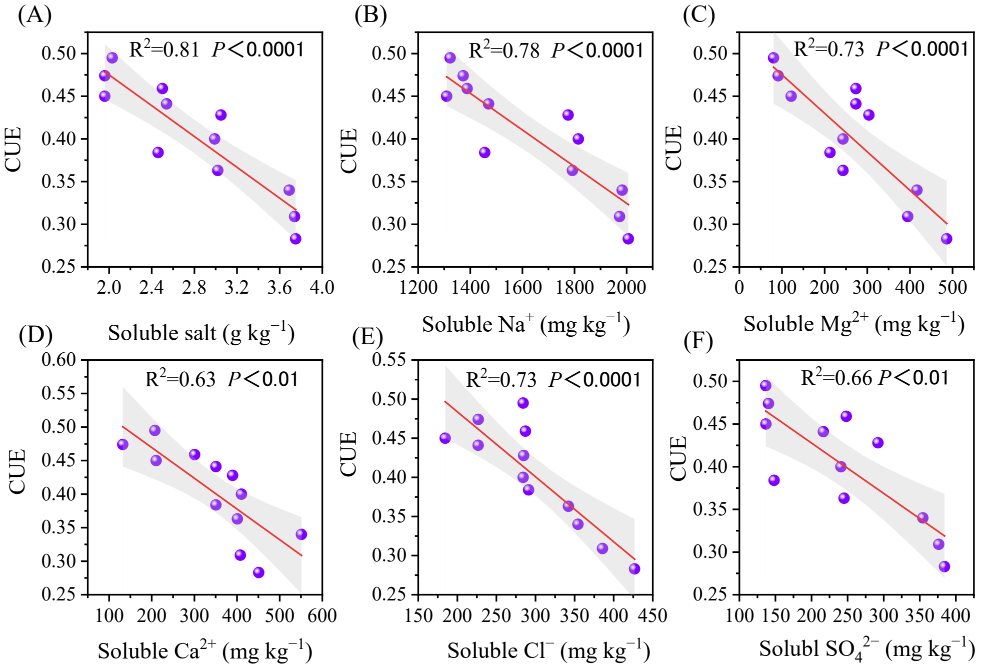

Figure S3. Relationships of the microbial carbon use efficiency (CUE) with total soluble salt (A), soluble Na^+ (B), soluble Mg^{2+} (C), soluble Ca^{2+} (D), soluble Cl^- (E), soluble SO_4^{2-} (F) in saline–alkali soil.

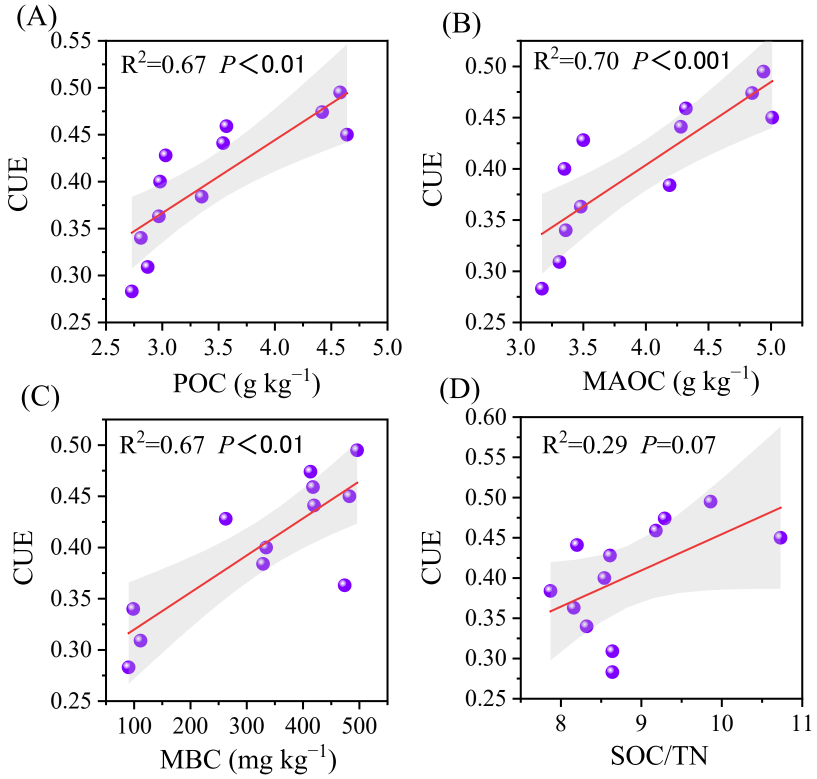

Figure S4. Relationships of the microbial carbon use efficiency (CUE) with particulate organic carbon (POC, (A)), mineral-associated organic carbon (MAOC, (B)), microbial biomass carbon (MBC, (C)), and SOC/TN (D) in saline–alkali soil.
